# Fixation devices made of poly-L-lactide composite for rib reconstruction after thoracotomy

**DOI:** 10.1186/s13019-024-02604-2

**Published:** 2024-03-15

**Authors:** Naoto Fukunaga, Hideki Sato, Tatsuto Wakami, Akio Shimoji, Otohime Mori, Kosuke Yoshizawa, Nobushige Tamura

**Affiliations:** 1 Department of Cardiovascular Surgery, Hyogo Prefectural Amagasaki General Medical Center, 2-17-77, Higashinaniwa-cho, Amagasaki, Hyogo 660-8550 Japan; 2 Research and Development Department, QOL Research Division, Gunze Medical Limited, Kyoto, Japan

**Keywords:** Bone fixation devices, Poly-L-lactide composite, Mesh-type plate and pin, Bioabsorbable fixation devices, Thoracotomy

## Abstract

GRAND FIX mesh-type plates and pins (Gunze, Kyoto, Japan) are thin, bioabsorbable fixation devices made of poly-L-lactide (PLLA) composite. These devices are new, and how best to use them in clinical practice remains to be determined. Thus, we describe our approach to rib fixation after thoracotomy in an aortic surgery using GRAND FIX mesh-type plates and pins. The advantage of our approach is that it is easy to perform, but careful attention should be paid to fix the ribs appropriately.

**Supplementary Information:**

The online version contains supplementary material available at 10.1186/s13019-024-02604-2.

## Introduction

Bone fixation devices made of hydroxyapatite and poly-L-lactide (HA/PLLA) composite are increasingly being used in the areas of thoracic surgery, orthopedic surgery, and trauma surgery [1–4]. Although authors have revised surgical techniques for rib fixation [1, 3, 4], a disadvantage of absent rotational stability still remains in thoracotomies when using pins to fix the ribs [1, 3]. It caused vertical or lateral displacements following the thoracotomies [3].

GRAND FIX mesh-type plates and pins (Gunze, Kyoto, Japan) are thin, bioabsorbable fixation devices made of PLLA composites. Regarding the process of the device production, PLLA is extended, enhanced by molecular orientation, and then molded into mesh-type plates or pins. PLLA is gradually degraded upon hydrolysis and absorbed in the human body. The polymer material causes minimal tissue reaction [5]. In animal models, safety of using PLLA pins to fix tibia fractures was reported, and an amount of PLLA degradation products were found at 3 years, which was faster compared with HA/PLLA devices between 4 and 6 years regarding the absorption in the body [6, 7]. Others demonstrated that PLLA sternal pins as an addition to regular sternal wires could increase the sternal stability under shear stress in juvenile swine models [8]. In clinical practice, PLLA pins were used to fix the sternum following cardiovascular surgery. Although the study did not show the efficacy to apply PLLA pins, there were no adverse events [9]. Efficacy of PLLA pins to fix the ribs has been published [5]. However, there has been no report as to how to use mesh-type plates as this device is new.

Here, we describe our approach to rib fixation after thoracotomy using GRAND FIX mesh-type plates and pins.

## Case report

**Fig. 1:**
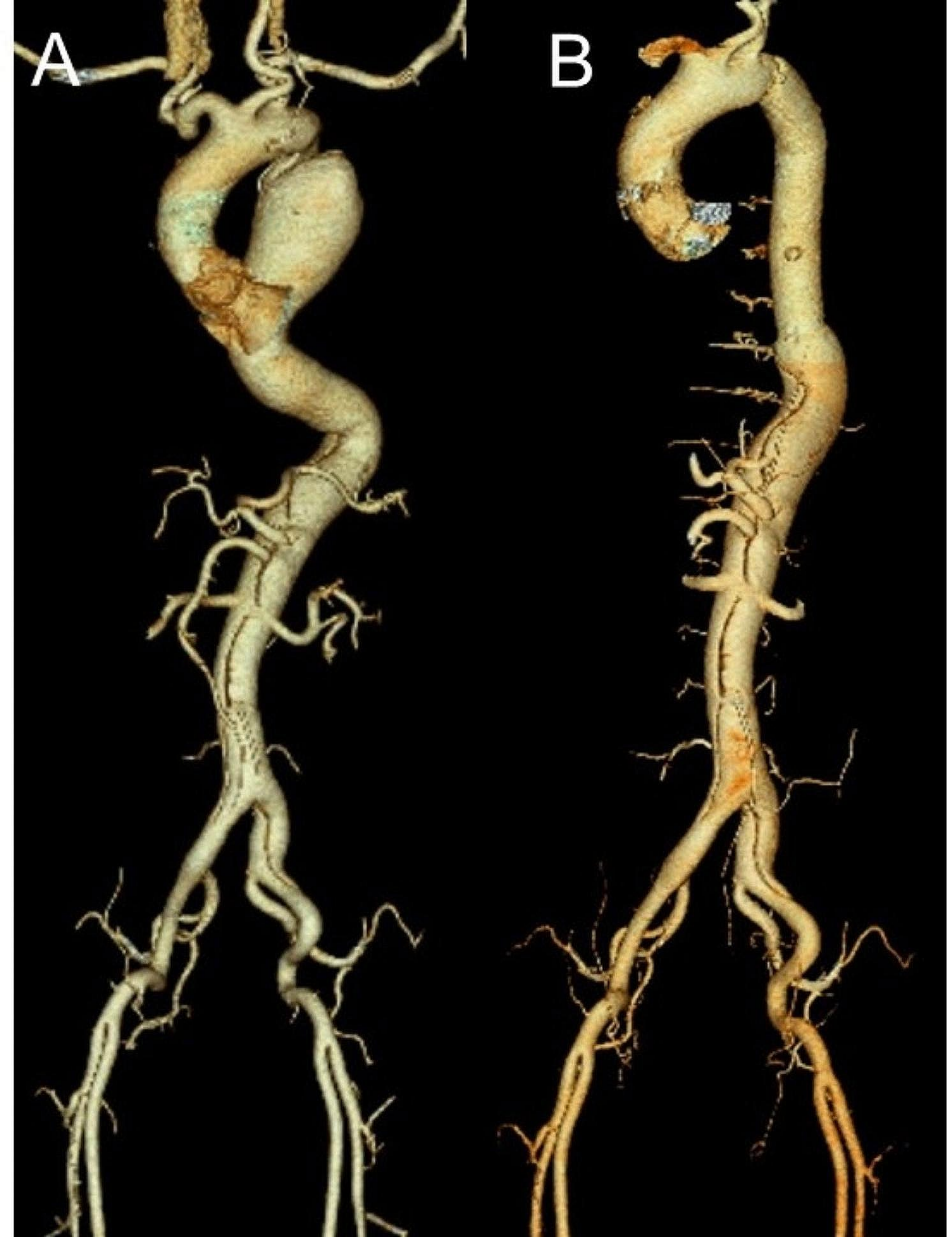
Preoperative computed tomography angiography reveals the dissecting aortic aneurysm in the descending aorta distal to the left subclavian artery (**A**) Postoperative computed tomography angiography shows replacement of the dissecting descending aorta with a vascular graft (**B**)

A 50-year-old man (height, 172 cm; weight, 84 kg) was referred to our hospital for an abnormal mass observed on chest X-ray. His medical history was untreated hypertension. Contrast-enhanced computed tomography (CT) revealed chronic dissecting aortic aneurysm in the descending aorta distal to the left subclavian artery (Fig. 1 [A]). The maximum diameter of the aneurysm was 6.6 cm, and the aneurysm terminated above the celiac trunk. There was no evidence of aortic rupture. The patient had never complained about symptoms. Blood work was within normal ranges. Transthoracic echocardiography showed normal left ventricular function without valvular issues. Cardiac CT confirmed that coronary arteries were normal.

The surgical approach was straight incision with rib-cross [10]. Three ribs were incised to enter the left thoracic cavity. The patient underwent graft replacement of the dissecting descending aorta under hypothermic circulatory arrest. Bilateral intercostal arteries at the level of Th8 were reconstructed.

**Fig. 2:**
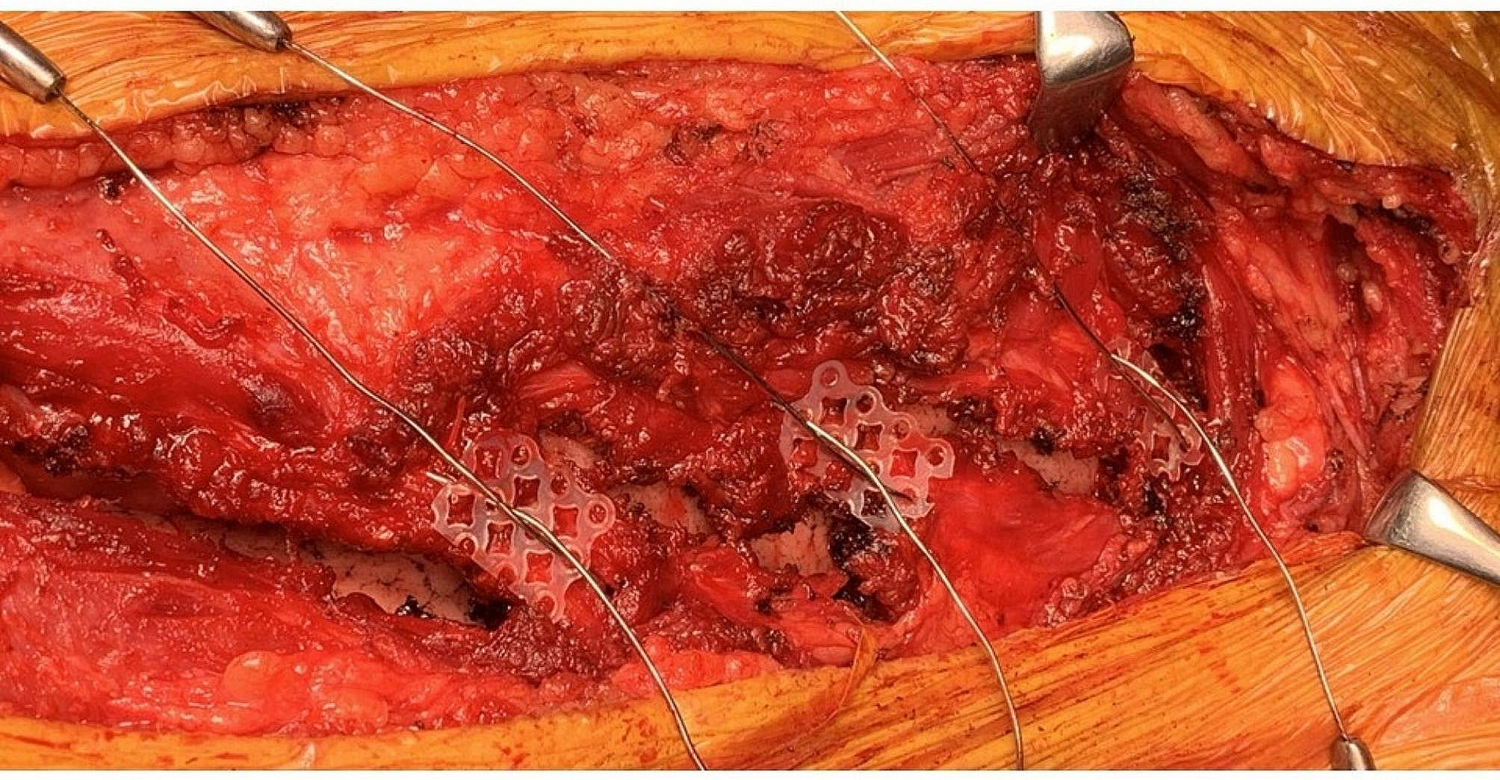
Intraoperative view just after crossing the sternal wires. All the incised ribs are brought together. The mesh-type plates are placed on the surface of the ribs

After meticulous hemostasis with protamine administration, the chest was closed. For each of the incised ribs, the bone marrow space was dilated with a dilator, and pins of appropriate-size were selected. A hand-drill was used to create a hole in each of the ribs on both sides of the cut to allow the passage of a sternal wire. The pins were inserted into the bone marrow of the incised ribs, and the two ends were brought together. A sternal wire was passed through the holes. A mesh-type plate was trimmed down to the width of each incised rib and placed on the ribs. Then, the sternal wire was tightened firmly with the mesh-type plate in-between the sternal wires to prevent rib breakage. On confirmation of sufficient approximation of the incised ribs (Fig. 2), the chest was closed in a standard fashion.

**Fig. 3:**
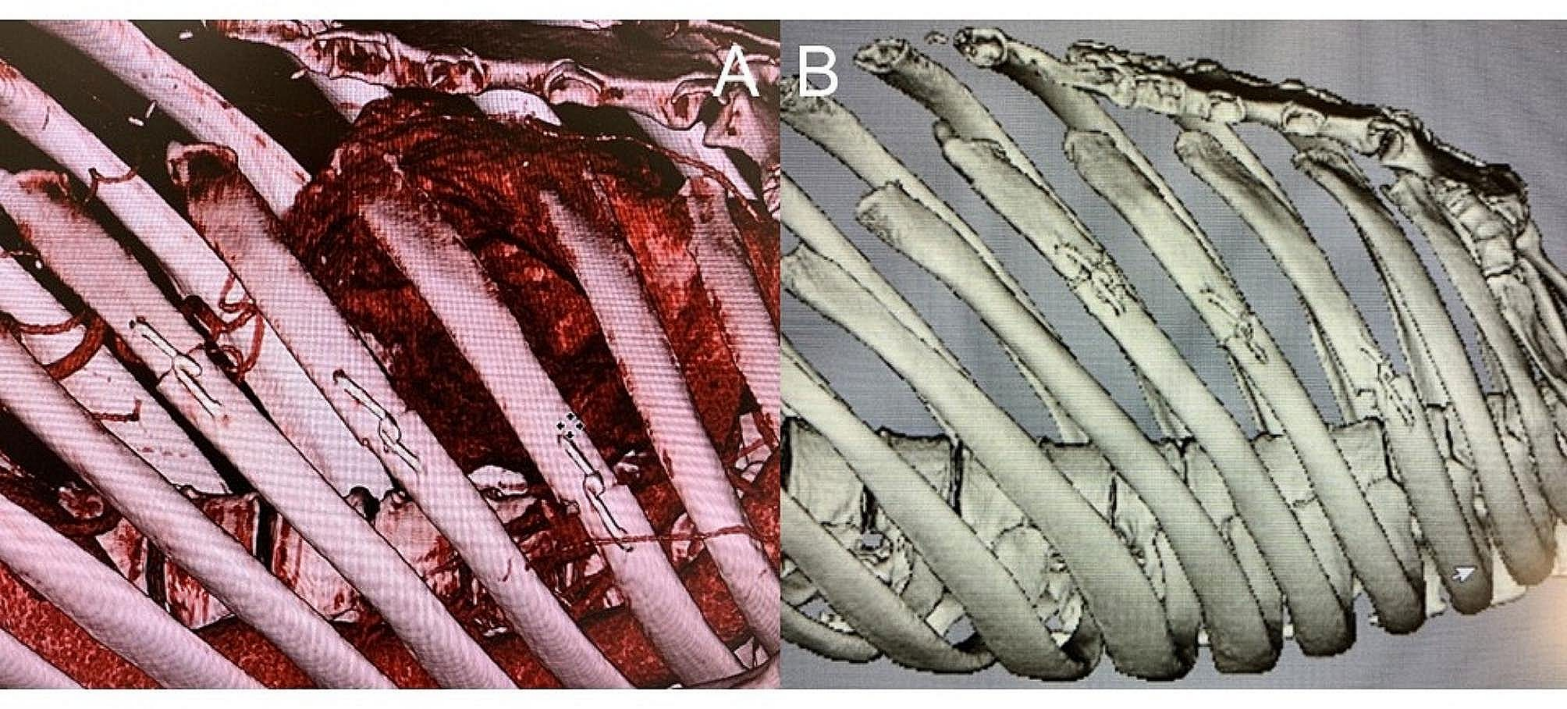
Computed tomography before discharge shows the fixed ribs (**A**) Computed tomography at eight weeks after surgery demonstrates fixed ribs. One incised rib is vertically displaced (**B**)

Postoperative three-dimensional CT showed replacement of the dissecting aortic aneurysm in the descending aorta with a vascular graft (Fig. 1 [B]) and no displacement of the fixed ribs (Fig. 3 [A]). No clinical symptoms such as local inflammation or infection were observed postoperatively. Cryoablation was performed to alleviate postoperative pain at the end of surgery, and the patient did not require any analgesic at discharge. His postoperative course was uneventful.

Follow-up CT at eight weeks after surgery showed that all three ribs had healed but also that one of the ribs was displaced (Fig. 3 [B]). The patient did not complain of the worsening pain.

## Discussion

Fixation devices made of HA/PLLA composite are increasingly being used in the areas of thoracic surgery, orthopedic surgery, and trauma surgery [1–4].

HA/PLLA composite devices are absorbable and can become fused to the bone by as soon as four weeks postoperatively, although complete absorption takes several years [11]. Regarding PLLA degradation, it takes about 3 years to become an amount of degradation products [6]. Experimental evidence has shown that fractures heal faster and stronger with absorbable plates compared with metal plates because metal plates prevent the bone from fully healing [5, 12, 13]. Furthermore, the low dynamic strength of PLLA and HA/PLLA composite materials has been shown to be beneficial for healing of fractured bones.

Costal coaptation pins made of HA/PLLA have been used by other groups to fix incised ribs [2, 3]. As authors described, the incidence of lateral, vertical, or combined displacement of ribs constructed following thoracotomy was over 30% at one year postoperatively. Although they noted a technical issue in how they approximated the ribs, their overall conclusion was that using HA/PLLA pins alone is ineffective for rib reconstruction [3]. Pins alone also do not appear to be effective for rotational fixation. Other group demonstrated the efficacy of PLLA costal coaptation pins with absorbable sutures to fix the ribs [5].

. Clinical assessment of efficacy of PLLA pins to fix the sternum following cardiovascular surgery found no cases of wound infection [9], while experimental study demonstrated that addition of PLLA pins could increase the sternal stability [8]. In the field of orthopedic and maxillofacial surgery, PLLA devices have been applied [5, 6]. The basic approaches for using PLLA devices are similar to those for using devices made of HA/PLLA composite.

To overcome the issues related to using pins alone, Ito et al. used mesh-type plates made of HA/PLLA composites in port-access cardiac surgery. Similarly, we used a mesh-type plate made of PLLA composite to prevent lateral and ventral displacement of ribs reconstructed after thoracotomy.

The mesh-type plates were placed on the ribs on both sides of the cuts, and fixed in place with sutures. Follow-up CT (at 19 weeks after surgery) showed that the ribs were held tightly in place by the plates and that no displacement had occurred [4].

Placing the plates on the surface of the ribs allowed us to bring the ribs tightly together by using sternal wires (Fig. 2). We did not place the mesh-type plate on the back of the ribs due to the risk of the edge of the plates causing mechanical lung injuries. Despite using the plates only on the front of the ribs, the CT scan at eight weeks showed that the ribs remained fixed. Given that the patient was a highly active individual with a large body size, we consider that the fixation was appropriate. We can expect fractured ribs to have healed by about three weeks; therefore, the reconstructed ribs in the patient had already healed with the aid of PLLA devices as a slight amount of PLLA degradation product was found at 3 years after implantation [6].

One issue in the present case was the vertical displacement of one of the reconstructed ribs that was found on the follow-up CT scan at eight weeks. We suspect that this displacement occurred just after surgery as a result of the rib not being properly approximated before chest closure. This finding indicates the importance of accurately assessing the fixation prior to chest closure. Regardless, our patient did not complain of the worsening pain during the follow-up period.

Here, we have described our approach to rib fixation after thoracotomy in which we use mesh-type plates and pins made from PLLA composite. Our approach is easy to perform, but careful attention should be paid to fix the ribs appropriately. We need to follow the patient to collect data. In addition, the larger study is needed to assess efficacy of a combination of PLLA mesh-type plates and pins.

### Electronic supplementary material

Below is the link to the electronic supplementary material.

Supplementary Material 1

## Data Availability

The datasets used in this case report are available from the corresponding author on reasonable request.

## Abbreviations

HA/PLLA: Hydroxyapatite and poly-L-lactide
PLLA: Poly-L-lactide
CT: Computed tomography
