# Supplementary material for: Fixation devices made of poly-L-lactide composite for rib reconstruction after thoracotomy
Source: J Cardiothorac Surg. 2024 Mar 15;19:130. doi: 10.1186/s13019-024-02604-2 (PMC10941403; doi:10.1186/s13019-024-02604-2)
Supplement: Supplementary file 1 — Supplementary Material 1 [file 13019_2024_2604_MOESM1_ESM.docx]

*Fixation devices made of poly-L-lactide composite for rib reconstruction after thoracotomy*

Naoto Fukunaga, Tatsuto Wakami, Akio Shimoji, Otohime Mori,

Kosuke Yoshizawa, Nobushige Tamura

Department of Cardiovascular Surgery,

Hyogo Prefectural Amagasaki General Medical Center, Hyogo, Japan.

Corresponding author:

Naoto Fukunaga, MD,

Department of Cardiovascular Surgery,

Hyogo Prefectural Amagasaki General Medical Center, Hyogo, Japan.

2-17-77, Higashinaniwa-cho, Amagasaki, Hyogo, Japan 660-8550.

Email: [naotowakimachi@hotmail.co.jp](mailto:naotowakimachi@hotmail.co.jp)

Abstract: 73 words

Total word count: 1512 words

**Abstract**

GRAND FIX mesh-type plates and pins (Gunze, Kyoto, Japan) are thin, bioabsorbable fixation devices made from poly-L-lactide (PLLA) composite. These devices are new, and how best to use them in clinical practice remains to be determined. Thus, we describe our approach to rib fixation after thoracotomy in an aortic surgery using GRAND FIX mesh-type plates and pins. The advantages of our approach are that it is easy to perform and it is reproducible.

**Introduction**

Bone fixation devices made of hydroxyapatite and poly-L-lactide (HA/PLLA) composite are increasingly being used in the areas of thoracic surgery, orthopedic surgery, and trauma surgery [1-4]. Although authors have revised surgical techniques for rib fixation [1,3,4], ~~there are some disadvantages that need to be overcome.~~ a disadvantage of absent rotational stability remains in thoracotomies when using pins to fix the ribs [1,3]. It caused vertical or lateral displacements following the thoracotomies [3].

GRAND FIX mesh-type plates and pins (Gunze, Kyoto, Japan) are thin, bioabsorbable fixation devices made from PLLA composites; however, because these devices are new, how best to use them in clinical practice remains to be determined.

Here, we describe our approach to rib fixation after thoracotomy using GRAND FIX mesh-type plates and pins. The advantages of our approach are that it is easy to perform and it is reproducible.

**Case report**

A 50-year-old man (height, 172cm; weight, 84kg) was referred to our hospital for an abnormal mass observed on chest X-ray. His medical history was untreated hypertension. Contrast-enhanced computed tomography (CT) revealed chronic dissecting aortic aneurysm in the descending aorta distal to the left subclavian artery. The maximum diameter of the aneurysm was 6.6cm, and the aneurysm terminated above the celiac trunk. There was no evidence of aortic rupture. The patient had never complained about symptoms. Blood work was within normal ranges. Transthoracic echocardiography showed normal left ventricular function without valvular issues. Cardiac CT confirmed that coronary arteries were normal.

The surgical approach was straight incision with rib-cross [5]. Three ribs were incised to enter the left thoracic cavity. The patient underwent graft replacement of the dissecting descending aorta under hypothermic circulatory arrest. Bilateral intercostal arteries at the level of Th8 were reconstructed.

After meticulous hemostasis with protamine administration, the chest was closed. For each of the incised ribs, the bone marrow space was dilated with a dilator, and pins of appropriate-size were selected. A hand-drill was used to create a hole in each of the ribs on both sides of the cut to allow the passage of a sternal wire. The pins were inserted into the bone marrow of the incised ribs, and the two ends were brought together. A sternal wire was passed through the holes. A mesh-type plate was trimmed down to the width of each incised rib and placed on the ribs. Then, the sternal wire was tightened firmly with the mesh-type plate in-between the sternal wires to prevent rib breakage. On confirmation of sufficient approximation of the incised ribs (Fig. 1), the chest was closed in a standard fashion.

Postoperative three-dimensional CT showed no displacement of the fixed ribs (Fig. 2 [A]). No clinical symptoms such as local inflammation or infection were observed postoperatively. Cryoablation was performed to alleviate postoperative pain at the end of surgery, and the patient did not require any analgesic at discharge.

Follow-up CT at eight weeks after surgery showed that all three ribs had healed but also that one of the ribs was displaced (Fig. 2 [B]).

**Discussion**

Fixation devices made of HA/PLLA composite are increasingly being used in the areas of thoracic surgery, orthopedic surgery, and trauma surgery [1-4].

HA/PLLA composite devices are absorbable and can become fused to the bone by as soon as four weeks postoperatively, although complete absorption takes several years [6]. Experimental evidence has shown that fractures heal faster and stronger with absorbable plates compared with metal plates because metal plates prevent the bone from fully healing [7,8]. Furthermore, the low dynamic strength of HA/PLLA composite materials has been shown to be beneficial for healing of fractured bones.

Costal coaptation pins made of HA/PLLA have been used by other groups to fix incised ribs [2,3]. As authors described, the incidence of lateral, vertical, or combined displacement of ribs constructed following thoracotomy was over 30% at one year postoperatively. Although they noted a technical issue in how they approximated the ribs, their overall conclusion was that using HA/PLLA pins alone is ineffective for rib reconstruction [3]. Pins alone also do not appear to be effective for rotational fixation.

There are currently no reports in the literature regarding the use of PLLA devices in clinical practice. However, the basic approaches for using PLLA devices are similar to those for using devices made of HA/PLLA composite.

To overcome the issues related to using pins alone, Ito et al. used mesh-type plates made of HA/PLLA composites in port-access cardiac surgery. Similarly, we used a mesh-type plate made of PLLA composite to prevent lateral and ventral displacement of ribs reconstructed after thoracotomy.

The mesh-type plates were placed on the ribs on both sides of the cuts, and fixed in place with sutures. Follow-up CT (at 19 weeks after surgery) showed that the ribs were held tightly in place by the plates and that no displacement had occurred [4].

Placing the plates on the surface of the ribs allowed us to bring the ribs tightly together by using sternal wires (Fig 1). We did not place the mesh-type plate on the back of the ribs due to the risk of the edge of the plates causing mechanical lung injuries. Despite using the plates only on the front of the ribs, the CT scan at eight weeks showed that the ribs remained fixed. Given that the patient was a highly active individual with a large body size, we consider that the fixation was appropriate. We can expect fractured ribs to have healed by about three weeks; therefore, the reconstructed ribs in the patient had already healed.

One issue in the present case was the vertical displacement of one of the reconstructed ribs that was found on the follow-up CT scan at eight weeks. We suspect that this displacement occurred just after surgery as a result of the rib not being properly approximated before chest closure. This finding indicates the importance of accurately assessing the fixation prior to chest closure.

Here, we have described our approach to rib fixation after thoracotomy in which we use mesh-type plates and pins made from PLLA composite. Our approach is easy to perform and reproducible.

**List of abbreviations**

**HA/PLLA:** hydroxyapatite and poly-L-lactide

**CT:** computed tomography

**Declarations**

**Availability of data and materials:**

The datasets used in this case report are available from the corresponding author on reasonable request.

**Competing interest:** None

**Funding:** None

**Authors contributions:** NF contributed to conception and design. NF wrote a manuscript. TW, AS, OM and KS participated in patient care and reviewed the manuscript. NT supervised the manuscript.

**Acknowledgements:** None

**Ethic approval and consent to participate:** Approval by the institutional review board was not required because of a nature of a case report. A consent form was obtained from this patient.

**References**

1. Nirula R, Diaz Jr. JJ, Trunkey DD, Mayberry JC. Rib fracture repair: indications, technical issues, and future directions. World J Surg 2009;33:14-22.

2. Dehghan N. Challenges in plate fixation of chest wall injuries. Injury, Int. J. Care Injuried 2018;49:S39-S43.

3. Kawachi R, Watanabe S, Suzuki K, Asamura H. Clinical application of costal coaptation pins made of hydroxyapatite and poly-L-lactide composite for posterolateral thoracotomy. Eur J Cardiothorac Surg 2008;34:510-513.

4. Ito T, Kudo M, Yozu R. Usefulness of osteosynthesis device made of hydroxyapatite-poly-L-lactide composites in port-access cardiac surgery. Ann Thorac Surg 2008;86:1905-1908.

5. Minatoya K, Seike Y, Itonaga T, Oda T, Inoue Y, Kawamoto N, et al. Straight incision for extended descending and thoracoabdominal aortic replacement: novel and simple exposure with rib-cross thoracotomy. Interact Cardiovasc Thorac Surg 2016;23:367-370.

6. Yasunaga T, Matsusue Y, Furukawa T, Shikinami Y, Okuno M, Nakamura T. Bonding behavior of ultrahigh strength unsintered hydroxyapatite particles/poly (L-lactide) composites to surface of tibial cortex in rabbits. J Biomed Master Res 1999;47:412-419.

7. Hanafusa S, Matsusue Y, Yasunaga T, Yamamuro T, Oka M, Shikinami Y, et al. Biodegradable plate fixation of rabbit femoral shaft osteotomies. a comparative study. Clin Orthop Relat Res 1995;315:262-271.

8. Viljanen J, Pihlajamaki H, Kinnunen J, Bondestam S, Rokkanen P. Comparison of absorbable poly-L-lactide and metallic intramedullary rods in the fixation of femoral shaft osteotomies: an experimental study in rabbits. J Orthop Sci 2001;6:160-166.

**Figure legends**

**Fig. 1** Intraoperative view just after crossing the sternal wires. All the incised ribs are brought together. The mesh-type plates are placed on the surface of the ribs.

**Fig. 2**

Computed tomography before discharge shows the fixed ribs (A).

Computed tomography at eight weeks after surgery demonstrates fixed ribs. One incised rib is vertically displaced (B).
